# Sudden Deaths in Puerperal State from Adynamic Lesions of the Nervous Centers, Etc.

**Published:** 1857-08

**Authors:** 


### SELECTIONS.

*Sudden Deaths in Puerperal State from Adynamic Lesions of the Nervous Centers, etc.* Translated by the Editors, from "L'Union Medicale de la Gironde de Bordeaux."

[CONTINUED.]

*2d. Pains.*—It is especially here that the most minute, anatomical and pathological investigations have failed. The female, in the puerperal state, may die suddenly, during or after *accouchement*, from pain or nervous exhaustion, resulting from a long and severe labor. There exists in the economy only a given sum of nervous force and power. If we consult the authors who have given special attention to this question, in connection with delivery, we find evident proofs of the fatal influence of a long labor, very painful and too prolonged. When the pain is violent, says Georget,* and when it persists a certain time, it provokes the contraction of the muscular system, it takes away all power of thought and throws the cerebral faculties promptly into an extreme collapse. A patient who dies from a major operation, when he has lost little or no blood, is stunned, overcome, and sometimes stupid; he is dejected, fatigued, broken down, and incapable of moving; he is pale; he is sometimes seized with an exaltation like to delirium, with loss of consciousness, disposition to vomit, and absolute vomiting, convulsive attacks, relaxations of the sphincters and involuntary dejections. Death has been the result of the pain. We attribute ordinarily the collapse of the cerebral faculties which follows the perception of the pain to a wasting of the sensibilty, a vague expression which explains the fact very badly and indeed does not explain it at all. We will obseve that this state is the effect of cerebral excitement, and that during the pain, as in all lively sensations and the affections less strong, the brain experiences a veritable super-excitation. Consecutively to these first accidents more or less grave are manifested in the brain or other organs. The pain and efforts of the labor with women predisposed to mental alienation by hereditary influence, by previous accessions, by an acute sensibility, produces, sometimes, alone, or by the aid of the most trifling cause, the development of this disease.

Not only, says Churchill, such a shock may be observed in certian labors, especially when they have been prolonged and severe, and produce, in this way, a sad result, but it exists, to a certain degree more or less marked, in almost all cases. It requires but little observation to recognize it. Thus, after an ordinary *accouchement*, the general sensibility is almost always excessive, although the senses may be more susceptible than ordinary, the eyes lose their brightness, and are feeble and languid; the least light is painful, as the slightest noise is offensive to the hearing; and if we do not give close attention to this excessive delicacy, very serious accidents may result. In ordinary cases, some hours of repose are sufficient to overcome this light collapse; but when the labor has lasted a very long

* Georget Dect. en 30, p. 499.

time, or when an operation, as version for example, has been nescessary, the phenomena are a great deal more pronounced. Pain, when it acquires a certain degree of intensity and duration, is in itself destructive. Difficult and prolonged labors become very often mortal from this cause; and even when there are no extraordinary difficulties, and when the labor is not too prolonged, a fatal prostration supervenes, whose explanation is only to be found in the pain. Delivery has been complete without any physical lesion, the woman has lost but the ordinary amount of blood from the uterine vessels, and in spite of the encouragement which she feels from her general condition and that of her infant, as well as from the conviction that her sufferings are at an end, does not regain her strength, nor her courage; but after an interval which does not exceed some hours, she falls into a state of prostration and sinking, and some hours afterward, in a manner quite unexpected and without any perceptible alteration, dies.*

Where is the physician, who has had any practice, who does not know the influence of a long and painful labor on the mother? At the moment of the accouchement, the uterine contractions succeed each other, and attain very soon a high degree of intensity known under the name of expulsive pains. Nature will very soon fulfill her end, if no obstacle intervenes to arrest the escape of the child; but if a contraction of either strait exists, a vicious position, a tumor, any obstacle whatever, the uterus will wear out against it all the power it has to furnish the labor, and then, exhausted with its vain efforts, it ceases entirely; but the child remaining in the uterine cavity, causes by its presence, new contractions, convulsive contractions, which determine, in the entire economy, a state of irritation and fever, followed by the most fatal accidents.

The face is then burning, the whole body is covered with sweat, the eye is fixed and haggered, its expression is sunken; the unhappy woman cries out, laments, begs for death, and calls out for some one to kill her, or put an end to her sufferings. The disturbance of the intellectual faculties, well marked, is sometimes complete, and they make, during their delirium, the most extravagant remarks. The cerebral excitation produced by the violence of the pain may end in insanity, and, in certain cases, physicians devoted to medico-legal medicine, have found, in this momentary disturbance of the intellect, the explanation of infanticides that all other circumstances left unintelligible.†

Observation 7th. A woman, whose pelvis was horribly deformed, was brought to us after the cranium had been perforated, and an attempt made to apply the crotchet. I made some tractions with M. Baudelocque. We employed, without success, all imaginable means. The cranium came by scraps at the point of the crotchets; it was necessary to stop, and during this moment of repose, the woman expired, retaining the child in the uterus. [*Memoires de Mad. La Chapelle*, *t.* 2, *p.* 227.] Those who devote themselves to the delicate and difficult part of *accouchements* in the midst of a great center of population, have more frequent occasion to observe these cruel terminations. I shall cite some examples. The two first were communicated by my father. The third came within my own observation.

---

*Travers' Inquiry 2d edition, p. 48.

† Cazeaux, p. 413.

Observation 8th. Mrs. X. of a good constitution; she is robust, 30 years of age; had, about two years ago, her first child. The child was delivered with forceps. At the second confinement, my father was called. No unusual symptom was present. The labor proceeded regularly during the first contractions; but when dilatation was complete, the head did not advance, and the woman exhausted herself with her vain efforts. In consultation, the application of the forceps was decided on. The introduction was easy, but tractions very sustained and continuous were necessary to bring away a voluminous and living child. The patient had suffered very much; she had expressed her sufferings in the most violent terms. The labor being over, she felt better, but overcome. The placenta was delivered a few instants after. The uterus contracted; the hemorrhage amounted to nothing. Very soon she manifested some contractions of the muscles of the face, the pulse became weak, and she expired suddenly. An autopsy was made.

Observation 9th. A woman in the country, of 28 years of age, in labor for two days, sent for us, with another confrère. We arrived at the house, and found a narrowing in the antero-posterior diameter of the superior strait. The forceps were applied in vain. The head, moveable and very high up, escaped from the forceps. Version was decided on, and was completed without very great difficulty, but the head remained a long time above the superior strait. By force of traction, it was expelled, and very soon the labor was finished; but at that very instant, she died—exhausted from pain,

In a notice on the presentation of the fact, read before you in 1850, I cited a case which terminated in the same fatal way.

Observation 10th. In 1848, I was sent for to see a lady 30 years old, who was in her second labor. M. Labayle was in attendance. The face presented excessive agitation, exaltation of the intellectual faculties difficult to be described, pains for more than eighty-four hours. On my arrival the pains were terrible, incessant, and each of them caused the patient to make frightful cries. Later, my father joined us, when the forceps were applied uselessly. Pelvic version was decided on. I accomplished it with the greatest trouble; and, after the most prolonged efforts I brought away a dead child. A half hour after she expired suddenly.

The following fact was communicated to the Gazette Medicalè, in 1837, by M. Villeneuve, of Marseilles:

Observation 11th. Ten days of pain, face pale, tongue dry and white, violent pain in epigastric region, uterus molded on fetus; after some repose, a bath, anodyne and excitant potion; forceps, but a single branch; craniotomy. The child was extracted, and the mother died two hours afterward.

Thus then, after, as during an accouchement, the fatal influence of effort and pain on the nervous system can not be mistaken. M. Cazeaux believes, with Churchill, that it consists in a more or less considerable shock of the cephalo-rachidien system. This shock is the result of the extraordinary disturbance produced by the parturition, and is in every respect like that produced by severe wounds, and from which the unfortunate workmen, whose extremities are crushed by machinery, sometimes die. This rapid and prompt form of death, which neither the circumstances of the accident, nor the lesions observed at the autopsy explain, is attributed by surgeons to the nervous shock. In all these cases, the nervous system plays a very important role; suffering greatly from shock, worn out by the sufferings of child-birth, the cerebro-spinal apparatus may often resist, but it has often a great deal of difficulty to recover from these successive shocks; it remains exhausted, without any power to awaken a last spark of life, and the autopsy does not teach us any thing.

The very excellent experiments, reported some years ago by M. Magendie, remove all doubts touching the influence of pain in the production of sudden death. If on a living animal we excite pains extremely acute, as for example, in piercing the posterior and spinal roots; if, previously, a graduated bent tube, containing mercury, has been introduced into the carotid artery, each painful sensation is marked by a moment of arrest in the contractions in the left ventricle, immediately followed by a repetition which carries the sanguine column higher. If these painful contractions are repeated too often, if the animal is weakened, a time arrives when a quick cessation of the contractions become definite: the animal is dead. In whatever point we excite pain, the result is the same."—*Cincinnati Medical Observer.*

